# Insights into the Time Evolution of Slowly Photodegrading Contaminants

**DOI:** 10.3390/molecules26175223

**Published:** 2021-08-28

**Authors:** Davide Vione

**Affiliations:** Department of Chemistry, University of Turin, Via Pietro Giuria 5, 10125 Turin, Italy; davide.vione@unito.it; Tel.: +39-011-6705296

**Keywords:** environmental photochemistry, direct photolysis, indirect photochemistry, recalcitrant pollutants, sunlight irradiance, seasonal variability, photochemical modelling

## Abstract

Photochemical degradation plays an important role in the attenuation of many recalcitrant pollutants in surface freshwaters. Photoinduced transformation kinetics are strongly affected by environmental conditions, where sunlight irradiance plays the main role, followed by water depth and dissolved organic carbon (DOC). Apart from poorly predictable weather-related issues, fair-weather irradiance has a seasonal trend that results in the fastest photodegradation in June and the slowest in December (at least in temperate areas of the northern hemisphere). Pollutants that have first-order photochemical lifetimes longer than a week take more than one month to achieve 95% photodegradation. Consequently, they may experience quite different irradiance conditions as their photodegradation goes on. The relevant time trend can be approximated as a series of first-order kinetic tracts, each lasting for one month. The trend considerably departs from an overall exponential decay, if degradation takes long enough to encompass seasonally varying irradiance conditions. For instance, sunlight irradiance is higher in July than in April, but increasing irradiance after April and decreasing irradiance after July ensure that pollutants emitted in either month undergo degradation with very similar time trends in the first 3–4 months after emission. If photodegradation takes longer, pollutants emitted in July experience a considerable slowdown in photoreaction kinetics as winter is approached. Therefore, if pollutants are photostable enough that their photochemical time trend evolves over different seasons, degradation acquires some peculiar features than cannot be easily predicted from a mere analysis of lifetimes in the framework of simple first-order kinetics. Such features are here highlighted with a modelling approach, taking the case of carbamazepine as the main example. This contaminant is almost totally biorecalcitrant, and it is also quite resistant to photodegradation.

## 1. Introduction

Photochemistry plays an important role in the environmental fate of contaminants in surface freshwaters, and particularly of those pollutants that are both biorecalcitrant and resistant to other abiotic transformation processes such as hydrolysis [1,2,3]. Photodegradation is usually divided into direct photolysis, where the contaminant absorbs sunlight and is consequently transformed, and indirect photoreactions [4]. In the latter case, sunlight is absorbed by compounds called photosensitisers (e.g., chromophoric dissolved organic matter (CDOM), nitrate and nitrite) to generate photochemically produced reactive intermediates (PPRIs). The main PPRIs are the hydroxyl radical, ^•^OH; the carbonate radical, CO_3_^•−^ and singlet oxygen, ^1^O_2_, as well as the excited triplet states of CDOM, ^3^CDOM* [5,6]. Pollutant indirect photodegradation is induced by reaction with PPRIs and, unlike direct photolysis, in indirect photochemistry pollutants do not need to absorb sunlight to be transformed [3].

Photodegradation kinetics of contaminants depend on their photochemical reactivity (absorption spectrum, direct photolysis quantum yield and second-order reaction rate constants with PPRIs) and on environmental parameters such as sunlight irradiance and spectrum, water depth and water chemistry (including the absorption spectrum of water, which is mostly accounted for by CDOM) [7]. The irradiance of sunlight is a major parameter that affects photodegradation; however, it varies with the time of the day and the day of the year, as well as with meteorological conditions [8]. Therefore, there is a need to define standard conditions of sunlight irradiance to obtain a definite photochemical lifetime for a given pollutant in a certain environment [9]. One often chooses summertime fair-weather conditions, to avoid issues connected with cloudy sky, and because photochemistry is most important when sunlight illumination is intense like in summer [7]. By so doing, one usually gets insight into the maximum photodegradation kinetics that a pollutant may experience in a given aquatic environment [10].

A step forward consists in describing photodegradation as a function of the month of the year. A typical approach is to take an average sunlight irradiance corresponding to the 15th day of the given month, which is a very reasonable approximation for the whole month [8]. By considering monthly variations in sunlight irradiance and spectrum, it is possible to highlight how photochemical kinetics change over different seasons. All other conditions being equal, photochemical lifetimes in winter can be longer by an order of magnitude compared to the corresponding lifetimes in summer [10].

However, an approach based on a month-by-month calculation of photochemical lifetimes works properly only if there is a reasonable chance for degradation to be completed within the given month. Assume the simple case of a pollution pulse followed by no emission afterwards (e.g., a spill), and assume that the pollutant undergoes photodegradation with first-order kinetics (C_t_/C_o_ = *e*^−k t^, where C_t_ is pollutant concentration at the time t, C_o_ the initial pollutant concentration, and k the pseudo-first-order photodegradation rate constant). Note that the reactions between pollutants and PPRIs are actually second-order ones, but PPRIs are in steady state and their concentrations can be considered constant. Therefore, these second-order reactions follow first-order (or better, pseudo-first-order) kinetics.

To achieve effective (say, 95%) photodegradation within the month, one needs C_t_/C_o_ = 0.05 at t = 30 days, which means that k should be at least 0.1 day^−1^. Note that k ~0.1 day^−1^ corresponds to a half-life time t_1/2_ = ln2 k^−1^ ~7 days, which is reasonably fast photodegradation kinetics. In fact, many pollutants will take longer to be photodegraded [11]. Although uniform first-order kinetics (day-and-night averaged) constitutes a good approximation within a single month, it is no longer valid if photodegradation takes longer than a month to complete under sunlight irradiance. In such a case a different approach should be used to describe the degradation trend, and several new issues will emerge that, to the author’s best knowledge, have not been addressed in detail before.

The first issue is understandably the calculation procedure to be used to describe photodegradation over time, in different months of the year. Because kinetics is much faster in summer than in winter [9], a reasonable conclusion would be that a pollution pulse is attenuated faster if it occurs in summer compared to winter. However, there is the problem of what happens if photodegradation is long enough that summer evolves into autumn, or winter into spring, as well as the behaviour of pollution pulses occurring in spring or autumn. Another potentially interesting question concerns the degradation of pollutants with different photochemical lability/stability, or even the same pollutant in different environments that might enhance or inhibit photodegradation [12].

The main goal of this work is to tackle the above issues by means of a modelling approach, to see if and to what extent the conclusions that may be drawn under standard sunlight irradiance conditions still hold in a more complex scenario. Photochemical modelling allows for considerable saving of time and resources compared to experimental or field studies, although it introduces unavoidable approximations. Moreover, in some cases the modelled scenarios might be excessively simplistic. However, there are indications that modelling may be sufficiently accurate [7] to enable the prediction of the photochemical behaviour of contaminants, in conditions that might be difficult to study by other methods.

For the sake of simplicity, it was assumed here that environmental conditions did not change over time, with the single exception of sunlight irradiance and spectrum. This assumption is reasonable for large lakes with long water retention times, where there is enough time for considerable photodegradation to take place before water leaves the lake [13,14]. Seasonal variations in photochemically important parameters (e.g., winter nitrate maxima and summer minima that depend on the biota, summer maxima of the dissolved organic carbon and summer minima of inorganic carbon and alkalinity due to CaCO_3_ precipitation) [15] were also neglected, to focus on the effect of irradiance alone. Therefore, the typical environment that is here described as a first approximation is a large oligotrophic lake with limited biological activity, where water is far from CaCO_3_ saturation. However, in a totally different scenario where water leaves a smaller lake, photodegradation will continue if the pollutant is still exposed to sunlight. The considerable complication in this case is that all the environmental conditions (water chemistry and depth, together with irradiance) change over time and should be taken into account. Moreover, changing conditions are clearly environment-specific and do not allow for general conclusions. For these reasons, it was assumed here that irradiance varied at constant water chemistry and depth.

## 2. Results and Discussion

To highlight the effects of photodegradation lasting over several months, one needs to consider recalcitrant pollutants. The selected compounds for photochemical modelling should undergo reasonably slow photochemical transformation, as well as negligible biodegradation or other pathways. Among the contaminants of emerging concern that are receiving considerable attention nowadays, acesulfame K and carbamazepine (CBZ) are known to be among the most recalcitrant [13,14]. Compared to acesulfame K, carbamazepine has been the subject of many more studies [16,17,18]. A more complete range of photochemical reaction parameters is available for CBZ [19] compared to acesulfame K, thus CBZ was chosen here as typical recalcitrant pollutant to model photodegradation kinetics (CBZ is mainly photodegraded by reaction with ^•^OH and ^3^CDOM*, plus a secondary role of direct photolysis [19]). In other circumstances there was the opposite need to highlight the effects of fast photodegradation, for which CBZ would hardly be suitable unless very favourable environmental conditions are considered. In these cases, diclofenac (DCF) was chosen as model photolabile compound, because it is known to undergo rather fast photodegradation in the natural environment [20,21].

**Figure 1:**
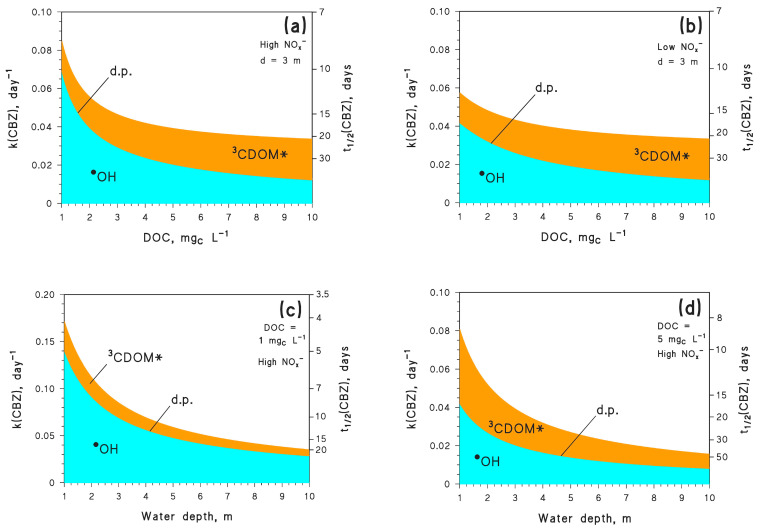
Modelled first-order degradation rate constants (k(CBZ)) and half-life times (t_1/2_(CBZ)) of CBZ, as a function of: (**a**) DOC, for water depth *d* = 3 m and high NO_x_^−^ conditions (10^−4^ M nitrate, 10^−6^ M nitrite); (**b**) DOC, for *d* = 3 m and low NO_x_^−^ (10^−6^ M nitrate, 10^−8^ M nitrite); (**c**) depth, for DOC = 1 mg_C_ L^−1^ and high NO_x_^−^; (**d**) depth, for DOC = 5 mg_C_ L^−1^ and high NO_x_^−^. In all cases it was assumed that [HCO_3_^−^] = 10^−3^ M and [CO_3_^2−^] = 10^−5^ M, with summertime (mid-July, mid-latitude) fair-weather irradiation. The importance of different photoreaction pathways is highlighted with different colours (d.p. = direct photolysis).

The first series of simulations was carried out with the APEX (Aqueous Photochemistry of Environmentally-occurring Xenobiotics) software [7], and had the goal of determining kinetics and pathways of CBZ phototransformation as a function of water depth, dissolved organic carbon (DOC), and the concentrations of NO_3_^−^ and NO_2_^−^ (NO_x_^−^). Figure 1 shows that the photodegradation of CBZ mainly occurs through ^•^OH and ^3^CDOM* reactions, with a negligible role of the other photochemical processes. Moreover, because [^•^OH] decreases and [^3^CDOM*] increases with increasing DOC [22,23], the relevant processes follow the same trend (Figure 1a,b). However, the inhibition of the ^•^OH photoreaction with increasing DOC is not offset by the enhancement of the ^3^CDOM* process, with the consequence that CBZ phototransformation becomes slower as the DOC gets higher. This is a rather common finding for aquatic photochemistry [10]. Moreover, given that NO_3_^−^ and NO_2_^−^ are both photochemical ^•^OH sources [5,24], it follows that [^•^OH] is higher at high NO_x_^−^ levels (NO_3_^−^/NO_2_^−^ = 100 µM/1 µM) compared to low NO_x_^−^ levels(NO_3_^−^/NO_2_^−^ = 1 µM/0.01 µM) (data not shown). Therefore, the reaction between CBZ and ^•^OH is faster, and the overall CBZ phototransformation is also faster, at high NO_x_^−^ (Figure 1a) compared to low NO_x_^−^ conditions (Figure 1b).

The photochemical lifetimes of CBZ obtained here by model calculations deserve a comment. A lifetime as low as, e.g., 10 days, might seem too low when compared with the persistence of CBZ observed in river water [25,26]. However, if river water flows with a reasonable value of linear flow velocity (1 m s^−1^) [27,28], after 10 days water has covered a distance of over 850 km. The first issue is that the vast majority of rivers are not that long. Moreover, even in the case of very long rivers one expects to find several wastewater treatment plants (WWTPs) over their course, each one being a likely source of CBZ. If two successive WWTPs are for instance separated by 100 km, with 1 m s^−1^ flow velocity and t_1/2_ = 10 days, one gets that only 7–8% CBZ emitted by the first WWTP would be photodegraded before river water mixes with the second WWTP outlet (C_t_/C_o_ = e^−k t^, k = ln 2 (t_1/2_)^−1^ = 0.07 day^−1^, and t = 1.1 days for water to cover 100 km). A 7–8% degradation percentage would be difficult to detect in field studies. Moreover, t_1/2_ = 10 days is quite short because it was obtained for CBZ at low DOC and under summertime irradiation conditions (Figure 1 reports considerably longer lifetimes in different conditions, even during summer). Therefore, it can be concluded that rivers are not suitable environments in which to highlight CBZ photodegradation, with the possible exception of very low flow conditions [11] that can be observed during severe water scarcity [28]. Large lakes might be more suitable environments to observe CBZ photodegradation.

Reaction kinetics slow down with increasing water depth (Figure 1c,d) because deep water bodies are not thoroughly illuminated by sunlight, unlike shallow waters [29]. High DOC conditions are again favourable to the ^3^CDOM* process but detrimental to overall CBZ phototransformation. Under the environmental conditions that are considered here, the CBZ photochemical lifetime in summertime is expected to vary from about one week to almost a couple of months. The overall results reported in Figure 1 suggest that depth and DOC are the water-body features that most affect photodegradation kinetics. In contrast, a variation by two orders of magnitude in the NO_x_^−^ levels only changes the first-order degradation rate constants and lifetimes of CBZ by ~25%.

**Figure 2:**
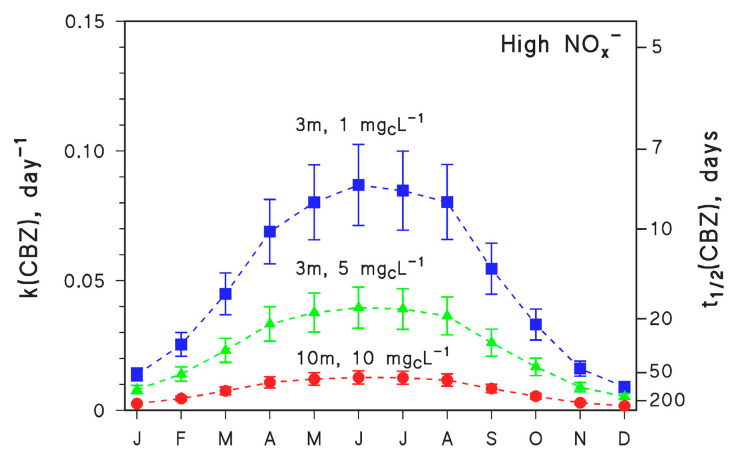
Modelled first-order degradation rate constants (k(CBZ)) and half-life times (t_1/2_(CBZ)) of CBZ as a function of the month of the year (45° N latitude, fair weather), for different values of water depth (*d* = 3 or 10 m) and DOC (1, 5 or 10 mg_C_ L^−1^). In all the cases, high NO_x_^−^ conditions (10^−4^ M nitrate, 10^−6^ M nitrite), [HCO_3_^−^] = 10^−3^ M and [CO_3_^2−^] = 10^−5^ M were assumed. The error bounds (μ ± σ) represent the uncertainties of the photochemical model.

Of course, summertime conditions are favourable to photodegradation [10], and the process is expected to slow down in different seasons. The seasonal trend of the kinetics of CBZ photodegradation is reported in Figure 2, for different values of the other environmental variables (water depth and DOC) that most affect photodegradation in addition to irradiance.

Photodegradation kinetics in fair weather follow the irradiance of sunlight, thus CBZ is expected to undergo the fastest degradation in June and the slowest in December (in temperate regions of the northern hemisphere). The ratio between the highest (June) and lowest (December) rate constants would be around 5–6 depending on conditions. It was mentioned before that t_1/2_ ≤ 7 days is needed for a compound to be completely photodegraded within one month. The simulation results reported in Figure 2 suggest that this would never be the case for CBZ under the considered conditions. Therefore, CBZ photodegradation is expected to span over several months.

The time evolution of CBZ was here modelled by assuming CBZ emission at the beginning of a given month, followed by photodegradation with month-dependent kinetics. The combination of a series of pseudo-first order degradation tracts, each lasting for one month, is described by Equation (1) (see Methods section for how this equation was obtained).

(1) $$C_{t}{/C}_{o}=\prod_{m=0}^{M}e^{-k_{m}\;{\Delta t}_{m}}$$

where *m* = 0 is the month of pollutant emission, *m* a generic month in the simulation, *M* the total number of months included in the simulation, k*_m_* the pseudo-first-order degradation rate constant of the contaminant in the month *m* (as returned by the APEX software), and Δt*_m_* = 28, 30 or 31 days. With these data, C_o_ is the initial contaminant concentration (*m* = 0) and C_t_ is the concentration at the end of the month *M*.

Water chemistry and depth were assumed not to vary over time, which is clearly an approximation but is supported by the fact that sunlight irradiance is more important than other environmental conditions in determining the degradation kinetics. Indeed, an examination of Figure 1 suggests that a variation in DOC by one order of magnitude (from 1 to 10 mg_C_ L^−1^) induced a twofold variation in the pseudo-first-order k(CBZ) (Figure 1a,b). However, seasonal DOC variations in a lake are very unlikely to go beyond a factor of two [30]. More important variations in k(CBZ) (up to fourfold, Figure 1c,d) were observed when water depth varied by an order of magnitude. However, water depth in a large lake is expected to vary much less than the DOC [30]. To halve the average depth of a lake, one needs exceptional water scarcity conditions such as the Millennium Drought in Australia, and rather shallow water column [31]. More extreme examples are the ephemeral lakes that completely dry out during the hot season, but they are quite rare environments [32]. Seasonal variations in [NO_3_^−^] may approach an order of magnitude [30], but Figure 1 suggests that even a variation by two orders of magnitude would have limited impact on k(CBZ). In contrast, seasonal variations of sunlight irradiance take place every year and produce important effects, with k(CBZ) seasonally varying by 5–6 times (Figure 2). Therefore, these results suggest that the assumption of variable irradiance at constant water chemistry and depth makes sense, even from an environmental point of view.

Another issue is that Equation (1) provides a time trend that reflects a single emission pulse followed by photodegradation. In the case of contaminants that are emitted continuously, it can still give insight into the fate of a compound that is discharged at a given time of the year. Field studies of course cannot go into such details, because compounds emitted at different times and not yet degraded are mixed together, but the results obtained by these simulations can still be informative.

**Figure 3:**
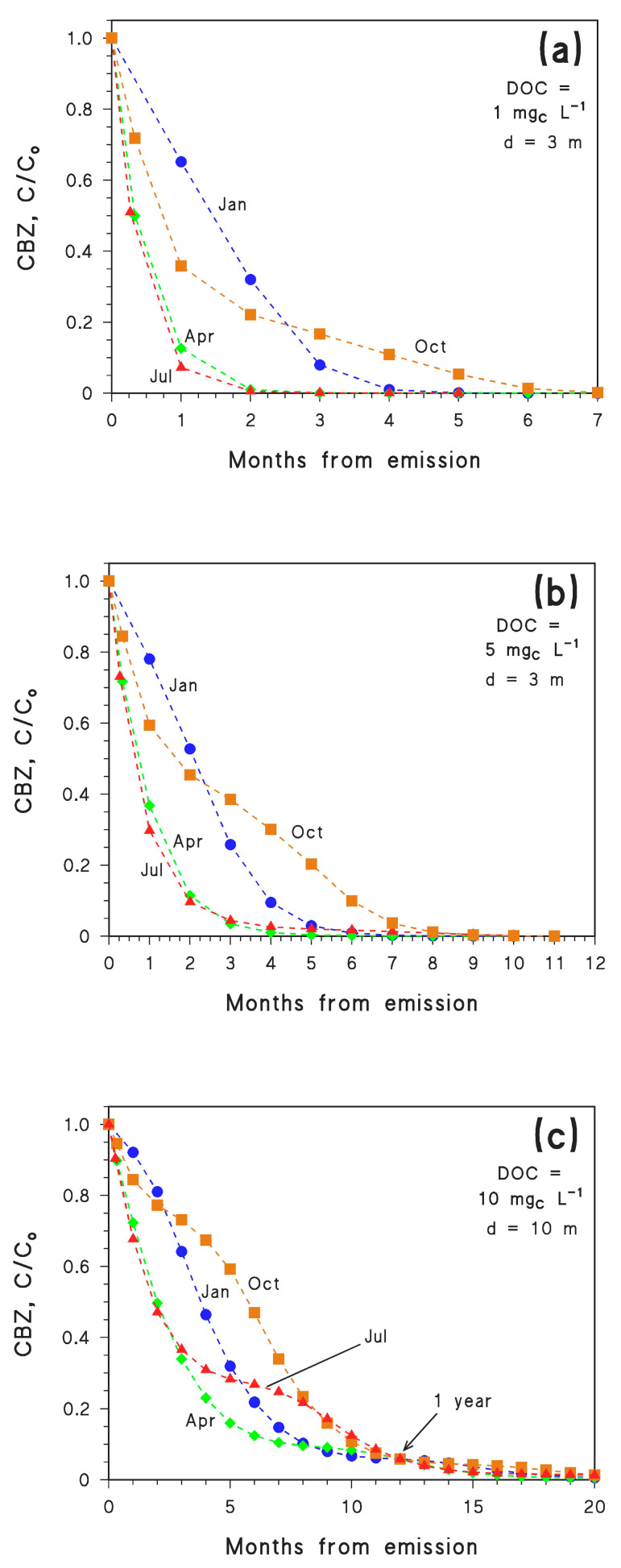
Time trends of CBZ (derived with Equation (1) and APEX simulations) in the different months following emission (the month of emission is highlighted near each time trend), for different conditions: (**a**) *d* = 3 m, DOC = 1 mg_C_ L^−1^; (**b**) *d* = 3 m, DOC = 5 mg_C_ L^−1^; (**c**) *d* = 10 m, DOC = 10 mg_C_ L^−1^. In all cases high NO_x_^−^ conditions (10^−4^ M nitrate, 10^−6^ M nitrite), [HCO_3_^−^] = 10^−3^ M and [CO_3_^2−^] = 10^−5^ M were assumed. Data points at 1 year are also highlighted in (**c**). Data points are connected by linear segments as a guide to the eye.

The simulation results obtained by application of Equation (1) are reported in Figure 3 for different values of the DOC and of water depth *d*. In particular, photochemical modelling refers to DOC = 1 mg_C_ L^−1^ and *d* = 3 m (Figure 3a), DOC = 5 mg_C_ L^−1^ and *d* = 3 m (Figure 3b), DOC = 10 mg_C_ L^−1^ and *d* = 10 m (Figure 3c). As already mentioned, CBZ photodegradation slows down with increasing depth and DOC (Figure 1). Therefore, it is not surprising to see that the conditions that most favour photodegradation are those reported in Figure 3a. According to the simulation results of Figure 3a, it can be seen that CBZ undergoes fast photodegradation if its emission takes place in spring (April) or summer (July), with very similar time trends in the two cases. Understandably, slower degradation is expected for emission in autumn (October) or winter (January), because the irradiance of sunlight is lower in these months. However, it should also be considered that CBZ photodegradation is too slow to be completed within one single month. Therefore, CBZ starts degrading faster when it is emitted in October compared to January, in agreement with the respective values of irradiance. However, as time progresses, on the one side degradation slows down as autumn evolves into winter, while in the other case one has an acceleration as winter evolves into spring. Interestingly, in the environmental conditions assumed for Figure 3a, emission in January would produce more effective photochemical elimination of CBZ compared to emission in October.

Similar considerations hold for DOC = 5 mg_C_ L^−1^ and *d* = 3 m (Figure 3b), although photodegradation is slower in these conditions.

The scenario represented by DOC = 10 mg_C_ L^−1^ and *d* = 10 m (Figure 3c) is the least favourable to CBZ photodegradation among the ones tested, which allows one to see how trends evolve at longer times. For instance, the trends relative to initial emission in April and July almost overlap in the first 3–4 months, but then the July curve slows down considerably as late autumn and winter are approached. Interestingly, one month after emission it can be seen that the concentration order is C/C_o_(July) < C/C_o_(April) < C/C_o_(October) < C/C_o_(January). In contrast, after seven months, C/C_o_(April) < C/C_o_(January) < C/C_o_(July) < C/C_o_(October). Another issue is that all the curves again reach the same C/C_o_ value after one year, which is understandable because in all the cases CBZ has experienced all the year-round sunlight conditions.

Furthermore, the October and January trends (the month always refers to initial emission) cross at around 2–3 months in all the cases (Figure 3a–c), almost independently of the environmental conditions and, as a consequence, of the degradation kinetics. A possible explanation is that this phenomenon mostly depends on sunlight exposure. The time trend data of CBZ concentration suggest that emission in October might be more favourable to degradation than emission in January, but only if complete degradation takes place in less than 2–2.5 months. The reverse is true if degradation takes longer, although differences between the two scenarios practically disappear at around 1 year. To get further insight into this behaviour, one needs a scenario with fast degradation kinetics.

**Figure 4:**
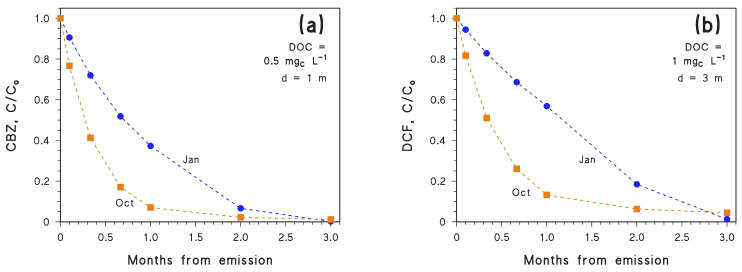
Time trends of (**a**) CBZ and (**b**) DCF, in the months following emission (the month of emission is highlighted). Assumed water conditions: [NO_3_^−^] = 10^−4^ M, [NO_2_^−^] = 10^−6^ M, [HCO_3_^−^] = 10^−3^ M and [CO_3_^2−^] = 10^−5^ M, plus (**a**) DOC = 0.5 mg_C_ L^−1^, *d* = 1 m, or (**b**) DOC = 1 mg_C_ L^−1^, *d* = 3 m. Data points are connected by linear segments as a guide to the eye.

For CBZ to be completely degraded in 2.5 months or less in autumn or winter, one needs lower DOC, lower depth, or both. Suitable conditions are DOC = 1 mg_C_ L^−1^ and *d* = 0.5 m, which would not be very common in the real environment. Figure 4a confirms that, under these very favourable environmental conditions for photodegradation, CBZ emitted in October would achieve effective removal faster than that emitted in January.

**Figure 5:**
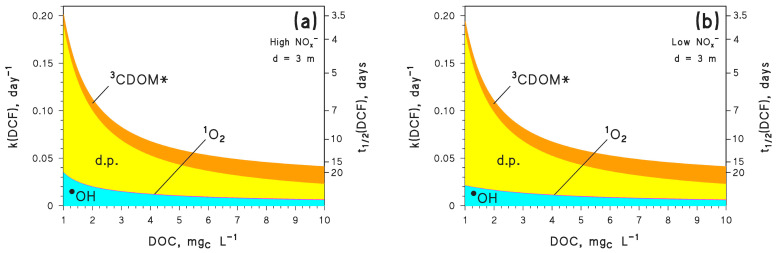
Modelled first-order degradation rate constants (k(DCF)) and half-life times (t_1/2_(DCF)) of DCF, as a function of DOC. Assumed water conditions: *d* = 3 m, [HCO_3_^−^] = 10^−3^ M and [CO_3_^2−^] = 10^−5^ M, plus (**a**) [NO_3_^−^] = 10^−4^ M, [NO_2_^−^] = 10^−6^ M, or (**b**) [NO_3_^−^] = 10^−6^ M, [NO_2_^−^] = 10^−8^ M. The importance of different photoreaction pathways is highlighted with different colours (d.p. = direct photolysis).

As an alternative, one should consider another contaminant that is degraded faster than CBZ. This is for instance the case for DCF which, unlike CBZ, undergoes effective degradation by direct photolysis as well [33]. Moreover, direct photolysis is the prevailing photodegradation pathway for DCF, except at very high DOC conditions where the ^3^CDOM* reaction can prevail (see Figure 5). As shown in Figure 4b, with DOC = 1 mg_C_ L^−1^ and *d* = 3 m, DCF emitted in October would be degraded faster than the DCF emitted in January. Another issue from Figure 4 is that the October and January trends cross again at around 3 months. Therefore, the phenomenon is not limited to CBZ and allows for the inference that emission in October, compared to January, would favour photodegradation of photolabile compounds but hamper photodegradation of more photostable ones. Indeed, when considering the same conditions (DOC = 1 mg_C_ L^−1^ and *d* = 3 m), it can be seen that CBZ would be eliminated faster if emitted in January (Figure 3a), while DCF would be eliminated faster when emitted in October (Figure 4b). This issue highlights the interplay between photochemical lability/stability of a pollutant and seasonality in defining the details of the photodegradation trend.

A further inference that can be derived by considering the results of photochemical modelling is that emission in April or July results in comparable photodegradation kinetics if degradation is completed within 3–4 months. In contrast, if photodegradation takes 5–9 months, the process is faster if initial emission takes place in April. The two scenarios become comparable again for a time scale of one year (Figure 3c).

**Figure 6:**
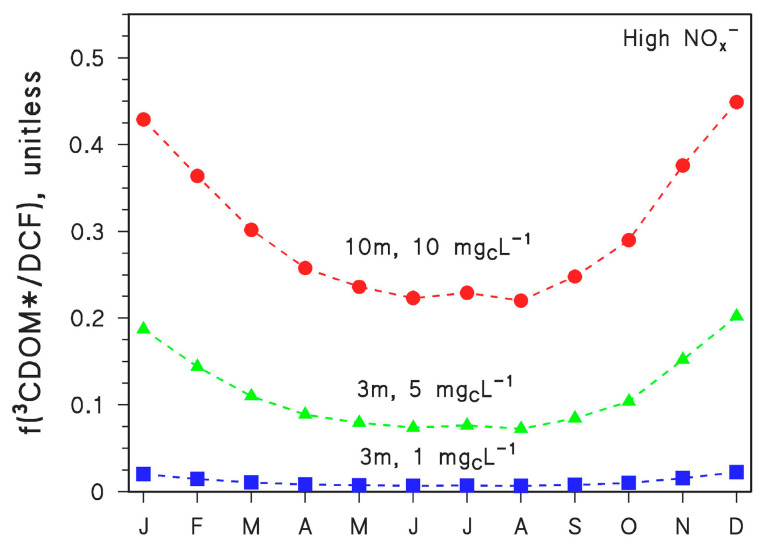
Modelled fraction of DCF phototransformation accounted for by ^3^CDOM* (f(^3^CDOM*/DCF)), in the different months of the year (45° N latitude, fair weather) and for different values of water depth (*d* = 3 or 10 m) and DOC (1, 5 or 10 mg_C_ L^−1^). In all cases, high NO_x_^−^ conditions (10^−4^ M nitrate, 10^−6^ M nitrite), [HCO_3_^−^] = 10^−3^ M and [CO_3_^2−^] = 10^−5^ M were assumed.

The photodegradation of CBZ yields mutagenic acridine (ACR) as the most concerning by-product. In particular, ACR is produced from CBZ with 3.1% yield by ^•^OH reaction and 3.6% yield by direct photolysis, while no ACR is formed from CBZ upon reaction with ^3^CDOM* [19]. The resulting, overall ACR yield from CBZ (*y*) depends on water conditions. Model calculations show that *y*~3% for *d* = 3 m and DOC = 1 mg_C_ L^−1^, *y*~2% for *d* = 3 m and DOC = 5 mg_C_ L^−1^, and *y*~1.3% for *d* = 10 m and DOC = 10 mg_C_ L^−1^. Seasonal variations in ACR yield are very small. In contrast, the most concerning DCF by-product is the aromatic amine 2,6-dichloroaniline (DCA), formed upon reaction between DCF and ^3^CDOM* (quantitative yield not available) [10]. Interesting, no DCA is formed from DCF direct photolysis or reaction with ^•^OH. Therefore, the fraction of DCF that is degraded upon reaction with ^3^CDOM* (f(^3^CDOM*/DCF)) is proportional to the overall DCA yield from DCF. The seasonal trend of f(^3^CDOM*/DCF) is reported in Figure 6 for *d* = 3 m and DOC = 1 mg_C_ L^−1^, *d* = 3 m and DOC = 5 mg_C_ L^−1^, and *d* = 10 m and DOC = 10 mg_C_ L^−1^. The reaction between DCF and ^3^CDOM* is enhanced at high DOC, because high-DOC waters also contain high CDOM, and irradiated CDOM is the source of ^3^CDOM* [5,7]. The ^3^CDOM* reaction is also enhanced in deep waters, because CDOM absorbs long-wavelength UV and visible radiation to a higher extent than nitrate and nitrite (^•^OH sources) and DCF itself (direct photolysis).

Indeed, the penetration in water columns is higher for long-wavelength radiation compared to short-wavelength radiation [5,7]. For the same reason that CDOM absorbs longer-wavelength radiation than other photosensitisers or DCF, the value of f(^3^CDOM*/DCF) is highest in winter and lowest in summer. Wintertime sunlight is particularly UVB-poor while summertime sunlight is UVB-rich [5,7]. As a consequence, DCF direct photolysis and ^•^OH reactions are especially inhibited during winter, when the relative importance of ^3^CDOM* in DCF degradation is, therefore, considerably higher.

## 3. Methods

**Figure 7:**
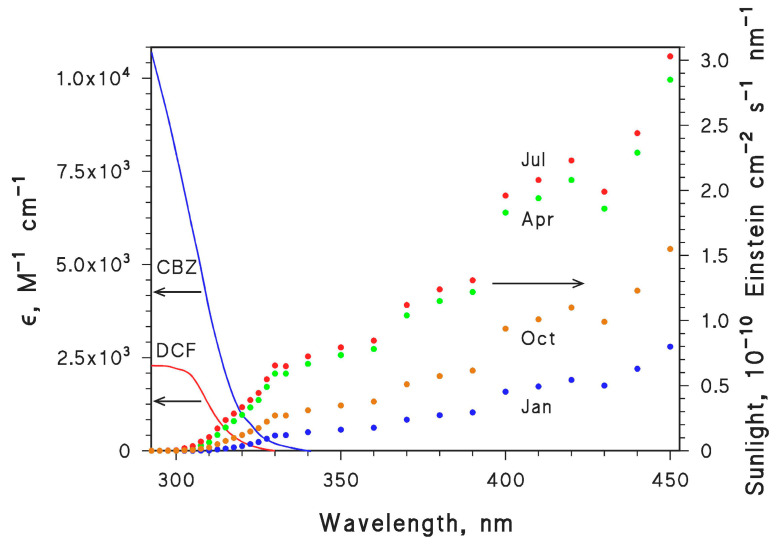
Left *y*-axis: Absorption spectra (molar absorption coefficients ε) of CBZ and DCF. Right *y*-axis: Spectral photon flux density of sunlight at 45°N latitude, in the months of January, April, July and October (15th day of each month).

The initial calculations of the first-order photodegradation rate constants of CBZ and DCF were carried out with the APEX (Aqueous Photochemistry of Environmentally-occurring Xenobiotics) software, available for free as Electronic Supplementary Information of [7,34]. This software predicts first-order photodegradation rate constants and half-life times based on photoreactivity parameters of contaminants (absorption spectra, direct photolysis quantum yields and second-order reaction rate constants with the PPRIs ^•^OH, CO_3_^•−^, ^1^O_2_ and ^3^CDOM*), and on data of water chemistry and column depth [7,34]. The choice of CBZ and DCF was also due to the fact that all the needed photochemical parameters are known for these compounds (see Table 1). Photochemical parameters, together with the absorption spectra of CBZ and DCF (shown in Figure 7) allow for the modelling of phototransformation reactions. Figure 7 also reports fair-weather sunlight spectra relative to January, April, July and October at 45° N latitude.

Moreover, APEX has been shown to reasonably predict the photochemical lifetimes of such contaminants in the environment. For instance, for CBZ in the epilimnion of Lake Greifensee (Switzerland) field data suggested t_1/2_ = 140 ± 50 days for photochemical reactions [16], while photochemical modelling predicted t_1/2_ = 115 ± 40 days [19] (interestingly, the authors of the field study provided a correction factor between fair-weather and actual meteorological conditions). In the case of Norra Bergundasjön (Sweden), field data suggested t_1/2_ = 1400 days (with confidence interval of 780–5700 days) [13], compared to 400–900 days predicted by the photochemical model [35]. Here a correction factor was not provided, which could partially account for the overestimation of photoreaction kinetics by the model. In the case of DCF, field data (Lake Greifensee) gave t_1/2_ = 8.3 ± 1.2 days [16], compared with t_1/2_ = 7.7 ± 0.8 days for model calculations [10].

APEX was thus used to provide first-order photodegradation rate constants (k) and half-life times (t_1/2_) of contaminants under summertime fair-weather conditions at 45° N latitude. The standard 24-h summertime day was 15 July. Then, photodegradation kinetics were calculated for different months by using the *APEX_season* function of the software, which carries out calculations for different values of sunlight irradiance at the same latitude. Uncertainties deriving from model parameters were determined based on error propagation, by means of the *APEX_error* function [34]. By so doing, one gets the monthly trend of photodegradation kinetics. Assume k*_m_* as the relevant first-order photodegradation rate constant in the month *m*, as calculated by *APEX_season*. In the pseudo-first-order approximation, the time trend of a contaminant (CBZ or DCF in this case) in the month *m* is given by the following equation:

(2) $$C_{t}=C_{o}\;e^{-k_{m}\Delta t}$$

where C_t_ and C_o_ were defined previously. For photodegradation over the whole month one should consider Δt = 28, 30 or 31 days, depending on the month *m*, which is a sufficiently short time span for the first-order approximation to hold.

If the pollution pulse occurs at the beginning of the month *m* and produces an initial pollutant concentration C_o_, by using the given value of Δt one can calculate C_t_, which is the concentration reached by the pollutant at the end of the month (in the hypothesis of absence of dilution).

If the pollutant is photolabile C_t_ may be low enough and, in this case, complete photodegradation may be reached within the month. However, in many cases photodegradation would be far from complete and would go on in the following month (*m* + 1). In this case, starting from C_t_ and using the value k*_m_*_+1_ returned by *APEX_season*, one can calculate the concentration $C_{t^{\prime }}$ at the end of the second month:

(3) $$C_{t^{\prime }}=C_{t}\;e^{-k_{m+1}\Delta t^{\prime }}=C_{o}\;e^{-k_{m}\Delta t}\;e^{-k_{m+1}\Delta t^{\prime }}$$

where, again, Δt′ = 28, 30 or 31 days depending on *m*+1. The last right-hand term of Equation (3) was obtained by substituting the value of C_t_ given by Equation (2) into the middle term of Equation (3). If another month is needed for additional photodegradation, one gets the following:

(4) $$C_{t^{\prime \prime }}=C_{t^{\prime }}\;e^{-k_{m+2}\Delta t^{\prime \prime }}=C_{o}\;e^{-k_{m}\Delta t}\;e^{-k_{m+1}\Delta t^{\prime }}\;e^{-k_{m+2}\Delta t^{\prime \prime }}$$

Briefly, the overall expression for the concentration of a contaminant due to photodegradation, month after month, can be expressed as follows:

(5) $$C_{t}{/C}_{o}=\prod_{m=0}^{M}e^{-k_{m}{\Delta t}_{m}}$$

where *m* = 0 is the month of pollutant emission, *m* a generic month in the simulation, *M* the total number of months included in the simulation, k*_m_* the pseudo-first-order degradation rate constant of the contaminant in the month *m* (as returned by *APEX_season*), and Δt*_m_* = 28, 30 or 31 days. With these data, C_o_ is the initial contaminant concentration (*m* = 0) and C_t_ is the concentration at the end of the month *M*.

In cases where photodegradation was relatively fast (DCF, as well as CBZ under the most favourable conditions) the initial time step of the simulation was chosen to be shorter than one month. By so doing, it was possible to produce a more accurate description of the photodegradation time trend.

## 4. Conclusions

Compounds that are photostable enough to have photochemical lifetimes longer than a week require more than one month to be (almost) completely removed. In the process they experience changing seasonal conditions of sunlight irradiance, even in the hypothesis of consistent sunny weather. As a consequence, photodegradation will depart from a simple first-order trend. This scenario has implications that differ from what can be derived by a mere analysis of photochemical lifetimes.

For instance, sunlight irradiance (and, as a consequence, the pseudo-first-order degradation rate constants of pollutants) are higher in July than in April. However, the irradiance evolution in the following months ensures that a compound emitted in April is photodegraded in almost exactly the same way as the same compound emitted in July, provided that effective removal is obtained within 3–4 months. If photochemical removal takes longer (but less than a year), emission in April allows for considerably faster elimination compared to emission in July.

Furthermore, for relatively photolabile compounds (effective elimination within 3 months), degradation is faster if they are emitted in October compared to January. However, the opposite happens for slower photodegradation. All the time trends join one another again at *t* = 1 year, because in a whole year a compound experiences all conditions of sunlight irradiance, independently of the month of emission.

These findings suggest that the photodegradation of relatively photostable compounds, which experience different conditions of sunlight irradiance while they are degraded has non-trivial implications, and that their time trend can no longer be approximated by uniform pseudo-first-order kinetics. It is shown here that, in contrast, the relevant time evolution can be reasonably approximated by considering a series of first-order tracts, each lasting for one month. Moreover, and counter-intuitively, emission during the summer season is not necessarily the most favourable scenario for slowly photodegrading pollutants, which might undergo more effective photodegradation if initially emitted during spring or winter. This issue suggests that there is a considerable difference in behaviour between fast and slowly photodegrading contaminants, which is linked to the seasonal variations in sunlight irradiance and spectrum.

## Figures and Tables

**Table 1:** Photochemical reaction parameters of carbamazepine (CBZ) [19], and diclofenac (DCF) [10]: direct photolysis quantum yield (Φ) and second-order reaction rate constants ($k_{\mathrm{PPRI},P}$ ) with the PPRIs ^•^OH, ^1^O_2_ and ^3^CDOM*. The reaction with CO_3_^•−^ can be neglected for both CBZ and DCF [10,19]. The direct photolysis quantum yields refer to irradiation under sunlight, which is mostly absorbed by CBZ in the UVA and by DCF in the UVB region.

	P = CBZ	P = DCF
Φ, unitless	7.8 × 10^−4^	9.4 × 10^−2^
kO•H,P, M^−1^ s^−1^	1.8 × 10^10^	9.3 × 10^9^
kO12,P, M^−1^ s^−1^	1.9 × 10^5^	1.3 × 10^7^
kC3DOM*,P, M^−1^ s^−1^	7.5 × 10^8^	6.4 × 10^8^

## Data Availability

Data used or produced in this work are provided by the author on request.

## Sample Availability

Samples of the compounds are not available from the authors.
